# Multi-ethnic validation of 15-item Geriatric Depression Scale in Chile

**DOI:** 10.1186/s41155-020-00146-9

**Published:** 2020-05-19

**Authors:** Lorena P. Gallardo-Peralta, Carmen Rodríguez-Blázquez, Alba Ayala-García, María João Forjaz

**Affiliations:** 1 School of Social Work, University of Tarapacá, 18 de Septiembre 2222, 1000000 Arica, Chile; 2 Faculty of Social Work, Complutense University of Madrid, Madrid, Spain; 3 National Epidemiology Centre, Carlos III Health Institute, Madrid, Spain; 4 Network Center for Biomedical Research in Neurodegenerative Diseases (CIBERNED), Madrid, Spain; 5 National School of Public Health, Carlos III Health Institute, Madrid, Spain; 6 Health Services Research on Chronic Patients Network (REDISSEC), Madrid, Spain

**Keywords:** Depression, Indigenous, Geriatric psychiatry, Psychometrics

## Abstract

**Background:**

There has been scant research published regarding the assessment of depression in ethnic groups, and few studies have addressed the validation of scales for standardized assessment of depressive symptoms among indigenous minorities.

**Objective:**

The aim of this study was to analyze the psychometric properties of the 15-item Geriatric Depression Scale (GDS-15) for a multi-ethnic sample of older Chilean adults.

**Methods:**

Cross-sectional study with a sample of 800 older people, 71% of whom were self-declared indigenous (Aymara/Mapuche).

**Results:**

The non-indigenous group had a higher total GDS-15 score and lower quality of life and wellbeing scores than the indigenous groups (*p* < 0.001). The GDS-15 had a KR-20 coefficient of 0.90 for the non-indigenous group, 0.80 for Aymara, and 0.85 for Mapuche. The homogeneity index was 0.38 for non-indigenous, 0.24 for Aymara, and 0.29 for Mapuche.

**Discussion:**

The GDS-15 showed satisfactory psychometric characteristics for the samples studied. However, the better results observed for the non-indigenous group suggest that some characteristics and content of the rating scale are not fully appropriate for the indigenous older population.

**Conclusions:**

There is a need to develop the transcultural validation of scales such as GDS-15, which are applied in a standardized manner in geriatric evaluations as part of primary healthcare.

## Introduction

Chile is one of the most aged countries in Latin America, with 19.3% of its population aged over 60 years (Ministry of Social Development, 2017). One of the main public health problems in Chile is the high incidence of depressive symptomology among advanced ages (Grundy et al., 2012). The prevalence rate is 23% among women and 12% among men, and these figures increase to 44.5% among older people with a high level of dependence (Sandoval, Tamiya, Lloyd-Sherlock, & Noguchi, 2016).

Certain groups have a higher propensity to suffer from depression, particularly in the case of indigenous ethnic minorities (McIntyre et al., 2017; Petribu, Lima, Brotto, Leao, & Arruda, 2017; Roh, Burnette, Lee, Lee, & Easton, 2016; Walker, Campbell, Dawson, & Egede, 2019). In the case of older Chilean adults, depression is more common among the indigenous population. This is confirmed by a study conducted with older indigenous (Aymara) and non-indigenous adults; using the standard GDS-15 cutoff for possible cases of depression (≥ 5), 40.1% of the Aymara sub-sample identified with depressive symptomatology (as opposed to 34.1% of the non-indigenous population) and the figure is higher in the case of Aymara woman, amounting to 47.8% (Gallardo-Peralta, Sánchez-Moreno, Barrón, & Arias, 2015). Indigenous adults have undoubtedly experienced adverse and stressful living conditions during their lifetimes that negatively affect their physical and mental health. Specifically, studies with indigenous Chileans report higher levels of illness, increased morbidity, and worse cognitive functioning (Mella, Alvear, Carrillo, & Caire, 2003; Oyarce & Pedreros, 2007).

Chile is characterized by its mainly mixed population; however, native indigenous communities are still maintained. Chilean indigenous communities represent 9% of the population and have historically experienced multiple oppressive, discriminatory, segregationist, and assimilationist processes (Gavilan, Vigueras, Madariaga, & Parra, 2018). After the ratification of ILO Convention 169, Chile began a State-led process of ethnic recognition that took the form of policies involving positive discrimination, protection, and even preservation of indigenous communities. Over the course of their lifetimes, older Chilean indigenous adults have been witnesses to these contradictory processes, often entailing conflict, relating to their indigenous ethnic status. Perhaps, partly as a result of these experiences, mental health is a risk factor among indigenous Chileans, and recent studies confirm an “ethnic health gradient”; in other words, these minorities are in a disadvantaged position in terms of health (Gallardo-Peralta, Sánchez-Moreno, & Rodríguez-Rodríguez, 2019). The two indigenous communities that participated in this research were the Aymara and the Mapuche; they are briefly contextualized below.

The Aymara people are descended from the *Tiwanaku* culture and hence continue to maintain frequent family and community exchanges with Bolivia and Peru. In Chile, the Aymara live in the far north of the country and their natural surroundings are the highland and valley areas of the foothills of the Andes. Their social organization is based on the *ayllu* community model and is highly family-focused. The villages and dwellings that they inhabit are isolated from the city and even from the rest of Chile, which has led in recent decades to depopulation and aging of native communities. Older adults who are aging in their native villages maintain certain cultural, social, and economic practices, such as the herding of camelids (llamas, guanacos). Interactions between older adults and members of their close and extended family tend to occur at religious festivals or other Aymara rites that are still celebrated in the villages, in which space older people play a leading role in cultural transmission.

The Mapuche people originally occupied areas from the center to the south of Chile and are currently concentrated in southern areas. The term Mapuche means “people of the land.” They were originally hunter gatherers. Mapuche organization distinguishes groups by geographical area, with *picunches* in the north, *huilliches* in the south, *pehuenches* in mountain areas, and *puelches* in the east. Geographically, they are resident in rural areas and connected to major cities; there has even been significant migration into the city. The Mapuche organization also revolves around the *lovche* community model and is highly family-focused. The older people who are still living in rural indigenous communities are dedicated to agricultural and farming activities. These people are the landowners, who provide them with status within their community, but they have also assumed responsibility for the care and upbringing of grandchildren due to the (temporary or permanent) migration of their children. These older people maintain various indigenous practices and tend to perform a leading role in ceremonies; this is directly related with the persistence of cultural activities.

### Present study

Evaluating depression among the older population requires the use of instruments that can be applied quickly, are easy to understand, and have suitable psychometric properties. The Geriatric Depression Scale (GDS) has these characteristics and is specifically applicable to geriatric populations (Chiang, Green, & Cox, 2009; Luo, Lou, Chen, & Chi, 2018). It has been translated into numerous languages and validated in various countries, including China (Chan, 1996), Spain (Martínez de la Iglesia et al., 2002), Colombia (Campo-Arias, Urruchurtu, & Solano, 2008), Portugal (Pocinho, Farate, Dias, Lee, & Yesavage, 2009), India (Kulathunga, Srikanth, Kathriarachchi, & De Silva, 2010), and Italy (Galeoto et al., 2018). As it is an instrument adapted for old age, the GDS has been applied to various pathologies (Chang-Quan, Bi-Rong, Zhen-Chan, Ji-Rong, & Qing-Xiu, 2010), including chronic pain (Turner, Ersek, & Kemp, 2005), chronic obstructive pulmonary disease (Omachi et al., 2009), and Parkinson’s disease (Massai et al., 2018).

The GDS is one of the most commonly used scales to identify depressive symptoms among indigenous ethnic minorities such as American Indians or Alaska Natives living in the USA (Roh et al., 2016), Xucurú in Brasil (Petribu et al., 2017), and Aymara in Chile (Gallardo-Peralta et al., 2015), but these studies do not report the validation process for this specific population. From the several existing short versions of the GDS, only the GDS-5 was validated for Chile non-indigenous population (Hoyl, Valenzuela, & Marín, 2000). So, the present study aims to validate the GDS-15 for a multi-ethnic sample of older Chilean adults, using both classic psychometric approach as well as Rasch analysis (RA), which allows seeing of the scale is invariant across specific groups such as by ethnicity.

## Methods

### Participants

The study followed a quantitative and transversal approach with a sample consisting of 800 older people living in the north and south of Chile. A sample stratified by sex, ethnicity, and place of residence (municipal or rural area) was used to ensure representativeness in each of these territories. The sample was therefore multi-ethnic and made up of two indigenous communities (369 Mapuche and 201 Aymara), in addition to participants who were not members of any indigenous ethnic group (231 people, or 29% of the sample).

Women represented 49% of the total sample (Table 1), while men were 51–54% of each ethnic group except for the Aymara, in which women made up 53% of the sample. The non-indigenous group was slightly older than the other groups, with 44% of participants in the 70–79 age range. For the Aymara and Mapuche respectively, 48 and 44% of the sample were in the 60–69 age group. Most of the sample was married (ranging from 48% for non-indigenous to 60% for Aymara participants). Education level was low for most of the sample: while 52% of non-indigenous participants had completed primary-level education, 63% of Aymara and 68% of Mapuche respondents had not.

**Table 1:** Participant characteristics by ethnic group

Variable	Categories	Non-indigenous (*n* = 231)	Aymara (*n* = 201)	Mapuche (*n* = 368)	Total
		*n* (%)	*n* (%)	*n* (%)	*n* (%)
Sex	Women	107 (46%)	106 (53%)	180 (49%)	393 (49%)
	Men	124 (54%)	95 (47%)	188 (51%)	407 (51%)
Age group	60–69 years	82 (35%)	97 (48%)	162 (44%)	341 (43%)
	70–79 years	102 (44%)	75 (37%)	134 (36%)	311 (39%)
	80+ years	47 (21%)	29 (15%)	72 (20%)	148 (18%)
Marital status	Married or cohabiting	112 (48%)	120 (60%)	202 (55%)	434 (54%)
	Single	47 (20%)	23 (11%)	54 (15%)	124 (15%)
	Widow	49 (21%)	45 (22%)	96 (26%)	190 (24%)
	Divorced, separated	23 (11%)	13 (7%)	16 (4%)	52 (7%)
Residence	North (region of Arica y Parinacota)	110 (48%)	201 (100%)	0	311 (39%)
	South (region of The Araucanía)	121 (52%)	0	368 (100%)	489 (61%)
Education	Primary school incomplete	56 (24%)	127 (63%)	250 (68%)	433 (54%)
	Primary school	120 (52%)	49 (24%)	76 (21%)	245 (31%)
	High school or vocational education	48 (21%)	21 (10%)	39 (10%)	108 (13%)
	Higher education	7 (3%)	4 (3%)	3 (1%)	14 (2%)

### Procedure

Trained professionals applied the face-to-face questionnaire via interviews conducted between August and October 2017. Spanish was the main language used for the scale. The native population uses Spanish on a daily basis, as it is the main language in Chilean society. However, the Aymara and Mapuche populations use their own languages in exclusively native interaction contexts. For this reason, the questionnaire included questions that use terms from the indigenous language. For example, the Aymara terms *usu*, *thani*, and *k'uyaña* and Mapuche (Mapudungun) terms *Kütrankülen*, *monguelen*, and *chañiukëlen* were used to word “I am unwell,” “health,” and “sadness,” respectively. The Ethics Committee of Tarapacá University and the National Council for Science and Technology of Chile approved and monitored the ethical aspects of the study. The adaptation of the Spanish version of the abbreviated GDS was authorized by the author (Martínez de la Iglesia et al., 2002). The scale is broadly used and available as free, open access, material (Ruipérez, 2009). All procedures performed in studies involving human participants were in accordance with the 1964 Declaration of Helsinki and its amendments or comparable ethical standards. The data were processed confidentially and anonymously, having first obtained the informed consent of participants.

### Measures

The measures used were GDS-15 (Brink et al., 1982), WHOQOL-OLD (Power, Quinn, Schmidt,, & WHOQOL-OLD Group, 2005), the De Jong-Gierveld Loneliness Scale (De Jong Gierveld & Van Tilburg, 2006), the Brief Resilient Coping Scale (BRCS) (Sinclair & Wallston, 2004), the Personal Wellbeing Index (PWI-8) (Cummins, Eckersley, Pallant, Van Vugt, & Misajon, 2003), and the Perceived Social Support Questionnaire (PSSQ) (Gracia, Herrero, & Musitu, 2002).

The GDS-15 is a 15-item scale using yes/no response options: 10 items indicate the presence of depression when answered positively and the rest (items 1, 5, 7, 11, and 13) imply depression when answered negatively. A cutoff has been proposed, with a score of ≥ 5 suggesting depression, and the following ranges indicating severity: 5–9 points for mild and 10–15 for moderate-to-severe depression.

The WHOQOL-OLD is a questionnaire used to assess quality of life specifically among older adults. It comprises a Likert scale consisting of 24 items with scores ranging from 1 to 5. The total score for the scale hence ranges from 24 to 120 points. This questionnaire has been validated among the Chilean population (Urzúa & Navarrete, 2013).

The abbreviated 6-item version of the De Jong-Gierveld Loneliness Scale (DJGLS-6) was used. The items are scored on a scale from 0 to 2, although they are subsequently recoded as dichotomous (0 or 1). The final score ranges from 0 (no loneliness) to 6 (extreme loneliness). This scale has not been validated among the Chilean population. However, it has been found to have appropriate psychometric properties among Peruvian adolescents and adults (Ventura-León & Caycho, 2017) and older Spanish adults (Ayala et al., 2012).

The BRCS is composed of 4 items with five Likert-scale response categories. Total scores range between 4 and 20. A total score equal to or lower than 13 indicates low resilience, while scores equal to or higher than 17 result in a classification of high resilience. The BRCS has not been validated among Chilean older people. However, the available evidence has reported the BRCS to be valid and reliable for Latin American older people (Caycho-Rodríguez et al., 2018).

The PWI is an 8-item scale relating to satisfaction and covering various aspects of quality of life. The overall value for the scale is obtained by adding together the items and then transforming the result into a score from 0 to 100. This scale has been validated for the Chilean population, including among indigenous older people (Gallardo-Peralta, Molina, & Schettini, 2019).

The PSSQ is a scale made up of 9 items evaluating the functional dimensions of support (emotional, advice, and assistance) and reciprocity of support with respect to each source. It also offers a total score for functional support and reciprocity of support, as well as the number of components of the network, and separate scores for the different sources of social support. The present study takes into account social support from spouse/partner, children, relatives (grandchildren, siblings, nephews, in-laws, etc.), and friends. This instrument is extensively used for the Spanish-speaking population, having been validated in various contexts (residential, general, hospital) (Herrero & Gracia, 2005). It has also been successfully used with older Chilean people, although it has not been validated among this population (Gallardo-Peralta et al., 2015).

### Data analysis

Descriptive statistics (frequency, mean, standard deviation, and range) were calculated for socio-demographic data and rating scale scores. All calculations were made using the sample as classified in the three ethnic groups: non-indigenous, Aymara, and Mapuche. The main variable, GDS-15 total score, did not fit normal distribution, and non-parametric statistics were therefore applied. Statistical differences between groups were ascertained with chi-square (with residual analysis and Bonferroni correction), Mann-Whitney, or Kruskal-Wallis tests, with post hoc analysis (Dunn test with Bonferroni correction) for the latter. The data were 100% fully computable, with no missing data.

The following psychometric properties of the GDS-15 were analyzed according to classical test theory: data quality and acceptability, internal consistency, and construct validity. The percentage of missing data for GDS-15 items and median, standard deviation, and minimum and maximum values of the total GDS-15 score were calculated for data quality and acceptability (McHorney & Tarlov, 1995). Internal consistency was explored by computing the corrected item-total correlation (criterion .20–.40), Kuder-Richardson Formula 20 (KR-20) coefficient (standard criterion > .70) used for dichotomous items, and item homogeneity (criterion > .30) (Aaronson et al., 2002). Convergent validity, a type of construct validity, was calculated using the Spearman’s rank correlation coefficients of GDS-15 total score with the other rating scales. Following the existing literature, a moderate correlation was hypothesized between GDS-15 and quality of life scales (*r*_S_ = .30–.50) (Juniper, Guyatt, & Jaeschke, 1996). Discriminant validity, another type of construct validity, was also explored by calculating the differences in GDS-15 scores in the sample grouped by variables of interest: sex, age group (60 to 69, 70 to 79, and 80 years and above), and ethnic group.

RA was performed to iteratively check for the following measurement attributes: fit to Rasch model, unidimensionality, reliability using the Personal Separation Index (PSI), and local item independence. The difference between the observed responses and the ones expected by the Rasch model was tested by an interaction chi-square test, with Bonferroni adjustment for the number of items. This chi-square reflects the invariance across the trait, and a non-significant difference is expected. Additionally, residual statistics should be within the ± 2.5 range.

RA also permits the checking of differential item functioning (DIF) by established groups such as sex, age, and ethnic group and comparing them through an analysis of variance. A random sample of 300 was used to minimize unnecessary modifications to the model (Linacre, 1994). RA with large sample sizes can highlight as statistically significant small deviations from the model at item level that otherwise would be well-fitting items. A detailed description of RA is available elsewhere (Martinez-Martin & Forjaz, 2012; Pallant & Tennant, 2007; Tennant & Conaghan, 2007).

All descriptive and classical psychometric analyses were performed using IBM SPSS Statistics 22, and RUMM2030 (Andrich, Sheridan, & Luo, 2010) was used for RA.

## Results

The descriptive statistics of the applied rating scales are displayed in Table 2. The GDS-15 total score was higher for non-indigenous participants: 4.72 as opposed to 2.69 and 3.81 for Aymara and Mapuche, respectively (*p* < .001). These differences remained statistically significant after controlling by age (*p* < .001). Quality of life and wellbeing rating scale scores were lower for the non-indigenous group (WHOQOL-OLD total 70.07; PWI 62.52) than for Aymara (76.69 and 78.52, respectively) and Mapuche (74.27 and 72.14, respectively) (*p* < .001).

**Table 2:** Descriptive statistics of applied rating scales

	Non-indigenous	Aymara	Mapuche	p*
	Mean (SD)	Mean (SD)	Mean (SD)	
**GDS total score**	**4.72 (4.31)**^**a**^	**2.69 (2.82)**^**a,b**^	**3.81 (3.56)**^**b**^	**< 0.001**
**WHOQOL-OLD**				
Sensory	10.10 (2.92)^a^	10.23 (3.22)^b^	11.17 (2.77)^a,b^	< 0.001
Autonomy	13.36 (3.03)^a,b^	14.69 (2.60)^a^	14.04 (3.09)^b^	< 0.001
Activities	12.19 (3.10)	14.39 (2.57)	13.69 (3.08)	< 0.001
Participation	11.91 (2.96)	14.24 (2.76)	13.60 (3.44)	< 0.001
Death	11.15 (5.04)^a,b^	9.39 (4.50)^a^	8.68 (3.82)^b^	< 0.001
Intimacy	11.36 (4.20)^a,b^	13.74 (3.51)^a^	13.09 (5.13)^b^	< 0.001
**Total**	70.07 (11.16)^a,b^	76.69 (9.68)^a^	74.27 (11.36)^b^	< 0.001
**DJGLS-6 total**	6.22 (3.11)^a,b^	4.58 (2.02)^a^	5.25 (2.49)^b^	< 0.001
**BRCS total**	14.12 (2.98)	14.54 (2.95)^a^	15.02 (3.22)^a^	< 0.001
**PWI total**	62.52 (14.65)	78.52 (15.09)	72.14 (17.09)	< 0.001
**PSSQ**				
Spouse/partner	3.10 (3.58)	2.94 (3.48)	3.20 (3.52)	0.503
Children	3.19 (3.38)^a,b^	5.27 (2.85)^a^	4.50 (3.25)^b^	< 0.001
Relatives	1.82 (3.01)^a^	2.27 (2.77)^a,b^	3.06 (3.40)^b^	< 0.001
Friends	2.51 (3.30)^a,b^	0.85 (2.08)^a^	1.24 (2.53)^b^	< 0.001

*DJGLS-6* de Jong-Gierveld Loneliness Scale, 6 item; *BRCS* Brief Resilience Coping Scale; *PWI* Personal Wellbeing Index; *PSSQ* Perceived Social Support Questionnaire

*Kruskal-Wallis test for the three groups, with Dunn-Bonferroni correction. Superscript letters indicate significantly different values

Applying the GDS-15 cutoff points, 38.9% of the non-indigenous sample had suspected depression, a significantly higher rate than among the Aymara and Mapuche groups (20.9% and 33.1%, respectively; see Table 3). There were also differences in the most frequently reported depressive symptoms by ethnic group. For the non-indigenous group, the most frequently reported items were not feeling full of energy (reported by 56.7% of participants) and dropping numerous activities and interests (55.0%). For the Aymara group, the most frequent items were getting bored (35.3%) and being afraid that something bad was going to happen (33.8%). Dropping numerous activities and interests (49.2%) and preferring to stay at home (47.8%) were the items most frequently reported by Mapuche participants.

**Table 3:** Frequency of people with problems in each GDS item and diagnosis of depression

Percentage of people with problems in each item	Non-indigenous		Aymara		Mapuche		
	***N***	%	***N***	%	***N***	%	***p***
1. Satisfied with your life? (No)	36	15.6^a^	10	5.0^b^	27	7.3^b^	< 0.001
2. Dropped many of your activities and interests? (Yes)	127	55.0^a^	54	26.9^b^	181	49.2^a^	< 0.001
3. Feel that your life is empty? (Yes)	47	20.3^a^	14	7.0^b^	49	13.3^a,b^	< 0.001
4. Get bored? (Yes)	96	41.6^a^	71	35.3^a^	75	20.4^b^	< 0.001
5. In good spirits most of the time? (No)	64	27.7^a^	25	12.4^b^	89	24.2^a^	< 0.001
6. Afraid that something bad is going to happen? (Yes)	96	41.6^a^	68	33.8^a,b^	96	26.1^b^	< 0.001
7. Feel happy most of the time? (No)	70	30.3^a^	28	13.9^b^	70	19.0^b^	< 0.001
8. Feel helpless? (Yes)	58	25.1^a^	27	13.4^b^	80	21.7^a^	0.009
9. Prefer to stay at home? (Yes)	123	53.2^a^	67	33.3^b^	176	47.8^a^	< 0.001
10. More problems with memory than most? (Yes)	36	15.6^a^	33	16.4^a^	110	29.9^b^	< 0.001
11. It is wonderful to be alive now? (No)	38	16.5^a^	11	5.5^b^	52	14.1^a^	0.001
12. Pretty worthless the way you are now? (Yes)	55	23.8^a^	18	9.0^b^	62	16.8^a^	< 0.001
13. Full of energy? (No)	131	56.7^a^	56	27.9^b^	145	39.4^c^	< 0.001
14. Feel that your situation is hopeless? (Yes)	60	26.0^a^	21	10.4^b^	82	22.3^a^	< 0.001
15. Most people are better off than you are? (Yes)	53	22.9^a,b^	37	18.4^b^	109	29.6^a^	0.009
**Diagnosis of depressive symptoms**							**< 0.001**
Normal (0–4 points)	141	61.0^a^	159	79.1^b^	246	66.8^a^	
Mild depression (5–9 points)	50	21.6^a^	36	17.4^a^	94	25.5^a^	
Moderate-to-severe depression (10–15 points)	40	17.3^a^	6	3.0^b^	28	7.6^b^	

Chi-squared test with Bonferroni correction. Superscript letters indicate significantly different values

The corrected item-total correlation coefficients are displayed in Table 4, with only item 15 in the Aymara group scoring below the threshold (.19). In general, these coefficients were higher for the non-indigenous group (range .21–.75) than for Aymara (.19–.59) or Mapuche (.29–.64). KR-20 was also consistently higher for the non-indigenous group (.90) than for Aymara and Mapuche (.79 and .85, respectively); this was also the case for the homogeneity index (.38, .24, and .29 for the non-indigenous, Aymara, and Mapuche groups, respectively).

**Table 4:** Internal consistency of GDS

Item-total corrected correlation GDS items	Non-indigenous	Aymara	Mapuche
1 R	0.521	0.280	0.589
2	0.521	0.258	0.425
3	0.677	0.498	0.494
4	0.614	0.416	0.528
5 R	0.669	0.595	0.585
6	0.444	0.351	0.410
7 R	0.740	0.554	0.636
8	0.734	0.589	0.556
9	0.498	0.327	0.292
10	0.206	0.273	0.374
11 R	0.670	0.403	0.537
12	0.749	0.575	0.509
13 R	0.531	0.569	0.424
14	0.642	0.482	0.518
15	0.565	0.186	0.509
**KR-20 coefficient**	**0.901**	**0.796**	**0.848**
**Item homogeneity**	**0.38**	**0.24**	**0.29**

*R* recoded (0➔1; 1➔0)

The GDS-15 correlated − .57 with PWI and − .56 with the BRCS for the non-indigenous population, with lower coefficients for Aymara (− .24 and − .24, respectively) and Mapuche (− .48 and − .40, respectively) (Table 5). GDS-15 correlation coefficients with the other rating scales were lower than .50 for all three ethnic groups. The GDS-15 did not show statistically significant differences by sex in the three groups. By age, the non-indigenous and Mapuche samples had higher GDS-15 scores in the oldest age group (80 years and above) (*p* < .05) than in the youngest age group.

**Table 5:** Convergent validity of the GDS-15 total score

	GDS-15 total, non-indigenous	GDS-15 total, Aymara	GDS-15 total, Mapuche
**WHOQOL-OLD** sensory	0.23**	0.12	0.15**
**WHOQOL-OLD** autonomy	− 0.47**	− 0.25**	− 0.39**
**WHOQOL-OLD** activities	− 0.54**	− 0.29**	− 0.34**
**WHOQOL-OLD** participation	− 0.59**	− 0.40**	− 0.46**
**WHOQOL-OLD** death	0.55**	0.27**	0.03
**WHOQOL-OLD** intimacy	− 0.48**	− 0.27**	− 0.21**
**WHOQOL-OLD total**	− 0.28**	− 0.19**	− 0.37**
**DJGLS-6 total**	0.36**	0.29**	0.28**
**BRCS total**	− 0.56**	− 0.29**	− 0.40**
**PWI total**	− 0.57**	− 0.24**	− 0.48**
**PSSQ spouse/partner**	− 0.14*	− 0.04	− 0.03
**PSSQ children**	− 0.18**	− 0.02	− 0.02
**PSSQ relatives**	− 0.01	− 0.00	0.06
**PSSQ friends**	0.16*	0.02	− 0.12*

Spearman’s rank correlation coefficients

*DJGLS-6* de Jong-Gierveld Loneliness Scale, 6 item; *BRCS* Brief Resilience Coping Scale; *PWI* Personal Wellbeing Index; *PSSQ* Perceived Social Support Questionnaire

*p < .05; **p < .01

Fit to the Rasch model was obtained after deleting items 7 (feel happy) and 10 (feel memory problems), which showed significant misfit. Residual fit was − 4.078 and 3.306 for items 7 and 10, respectively, both with a significant chi-square interaction (*p* = .000002 and *p* = .000439). The PSI was .68, there was local item independence, the scale was unidimensional, and there was absence of DIF for all items except for item 4 (often get bored). For the same depression level, non-indigenous people tended to answer item 4 at a higher level, and Mapuche at a lower level than Aymara. There was no DIF by sex or age.

## Discussion

The aim of the present study was to analyze the psychometric properties of the GDS-15 for a multi-ethnic sample of older Chilean adults, specifically among two indigenous communities (Aymara and Mapuche) and among participants not identifying as indigenous. The results of this study indicate that the psychometric properties of the GDS-15 are acceptable for the groups analyzed. However, the GDS-15 shows higher internal consistency among the non-indigenous Chilean population. This result indicates the importance of creating geriatric assessment scales that are culturally adapted for ethnical and indigenous minorities (Almeida et al., 2014). The GDS-15 can be culturally adapted to the indigenous communities subject to study. For example, the expressions correlating to “dropped,” “helpless,” and “hopeless” could be replaced by their respective indigenous terms.

Notwithstanding this, the greatest difficulty to be taken into account is the fact that the concept of wellbeing and general health in the indigenous communities analyzed—*suma qamaña* and *küme mongen*—is the result of a balancing process involving individual/spiritual, social, and community aspects (Guerrero, 1995). The GDS-15 is a scale for the assessment of depressive symptoms that is focused on individual processes, but it does not take into account the importance of community in the subjective aspects of depression. To overcome this, clinicians should have knowledge of their patients’ socio-cultural context and implement comprehensive psychogeriatric interviews (Hunter, 2014).

The results of this study also suggest differences in the prevalence of depressive symptoms depending on ethnic group. Unexpectedly, 85.6% of the Aymara group did not present depressive symptoms, despite previous studies identifying a higher incidence of this neuropsychiatric disorder among this indigenous group (Gallardo-Peralta et al., 2015). Being resident in native communities and natural environments may act as a protective factor in this regard. However, the same results were not observed for the Mapuche community, with 16.8% being classified in the moderate depression category. This indigenous Chilean community has encountered and continues to experience major problems with the State of Chile and is in a situation of greater social exclusion (Pacheco, 2012). Finally, 17.3% of the non-indigenous group presented severe depression, data that invite consideration of the factors that may negatively affect health among older non-indigenous adults living in areas that historically belonged to the indigenous population of Chile.

Despite ethnic groups presenting significant differences in the level of depression measured by the GDS-15, RA showed absence of DIF for virtually all items. These results are not contradictory. Rather, they indicate that GDS-15 items are not biased by ethnicity, with the exception of item 4 (feel happy). To account for DIF of item 4 by ethnicity, specific scores may be developed for each group.

Congruently with previous Rasch studies, our results also support the scale’s unidimensionality, local item independence, and fit to the Rasch model after deleting items 7 and 10. A Rasch analysis performed in another South American country, Brazil, also revealed misfit of these two same items (Chachamovich, Fleck, & Power, 2010). Likewise, item 10 showed misfit for a US sample of older adults (Chiang et al., 2009). Results for these two items should be interpreted cautiously.

With respect to convergent validity, the strongest association is with WHOQOL-OLD and there are lower correlations with the Loneliness Scale and PSSQ. Again, these results show the need to culturally adapt instruments that have been standardized for the general non-indigenous population.

There were no missing data in this study for the various GDS-15 items assessed, which is due to the quality control process in the face-to-face interviews carried out by social and health science professionals who were qualified in conducting interviews among indigenous groups with low levels of education prior to carrying out the fieldwork.

This study was subject to certain limitations. First, the study design is cross-sectional. This invites a cautious interpretation of the potential existence of causal relationships. Second, although the sample was representative for the regions in the north (Arica and Parinacota) and south (Araucanía) of Chile, it is not a national sample. The results cannot therefore be generalized for all Chilean older people and should be understood and applied in the specific geographical context in which they were collected. The final limitation is not having reference points to compare the potential diagnosis of depression in indigenous communities.

Despite these limitations, the use of both CTT and RA methodologies offers added value to this study by providing robust psychometric results. This study hence represents one of the first validations of the GDS-15 among Latin American indigenous communities living in native territories.

## Conclusion

In conclusion, the GDS-15 is generally an acceptable, reliable, and valid instrument to assess depression among Chilean older people, including ethnic minorities. With small modifications, it shows good measurement properties according to the Rasch model, including the absence of bias by ethnic group.

A comparison of validation results for the indigenous and non-indigenous samples suggests a need for geriatric rating scales that take into account the specific characteristics of indigenous older adults. Using this kind of instrument would enable researchers and clinicians to detect depressive symptoms and other signs of mental illness, in order to prevent such illnesses and implement appropriate interventions to address the needs of indigenous geriatric populations.

## Data Availability

Data can be provided upon justified request to the first author.

## Abbreviations

BRCS: Brief Resilient Coping Scale
CTT: Classical Test Theory
GDS-15: Geriatric Depression Scale-15 items
PSSQ: Perceived Social Support Questionnaire
PWI-8: Personal Wellbeing Index-8 items
RA: Rasch analysis
WHOQOL-OLD: World Health Organization Quality of Life-Old

## References

[CR1] Aaronson N, Alonso J, Burnam A, Lohr KN, Patrick DL, Perrin E, Stein RE (2002). Assessing health status and quality-of-life instruments: attributes and review criteria. Quality of Life Research.

[CR2] Almeida OP, Flicker L, Fenner S, Smith K, Hyde Z, Atkinson D (2014). The Kimberley assessment of depression of older Indigenous Australians: prevalence of depressive disorders, risk factors and validation of the KICA-dep scale. PLoS One.

[CR3] Andrich D, Sheridan B, Luo G (2010). RUMM2030 (Computer software and manual).

[CR4] Ayala A, Rodríguez-Blázquez C, Frades-Payo B, Forjaz MJ, Martínez-Martín P, Fernández-Mayoralas G, Rojo-Pérez F (2012). Psychometric properties of the Functional Social Support Questionnaire and the Loneliness Scale in non-institutionalized older adults in Spain. Gaceta Sanitaria.

[CR5] Brink TL, Yesavage JA, Lum O, Heersema PH, Adey M, Rose TL (1982). Screening tests for geriatric depression. Clinical Gerontologist.

[CR6] Campo-Arias A, Urruchurtu Y, Solano T (2008). Consistencia interna, estructura factorial y confiabilidad del constructo de la Escala de Yesavage para depresión geriátrica (GDS-15) en Cartagena (Colombia). Revista Salud Uninorte.

[CR7] Caycho-Rodríguez T, Ventura-León J, García-Cadena CH, Tomás JM, Domínguez-Vergara J, Daniel L, Arias-Gallegos WL (2018). Psychometric evidence of a brief measure of resilience in non-institutionalized Peruvian older adults. Psychosocial Intervention.

[CR8] Chachamovich E, Fleck MP, Power M (2010). Is Geriatric Depression Scale-15 a suitable instrument for measuring depression in Brazil? Results of a Rasch analysis. Psychology Health and Medicine.

[CR9] Chan AC (1996). Clinical validation of the Geriatric Depression Scale (GDS): Chinese version. Journal of Aging and Health.

[CR10] Chang-Quan H, Bi-Rong D, Zhen-Chan L, Ji-Rong Y, Qing-Xiu L (2010). Chronic diseases and risk for depression in old age: a meta-analysis of published literature. Ageing Research Reviews.

[CR11] Chiang KS, Green KE, Cox EO (2009). Rasch analysis of the Geriatric Depression Scale-Short Form. The Gerontologist.

[CR12] Cummins RA, Eckersley R, Pallant J, Van Vugt J, Misajon R (2003). Developing a national index of subjective wellbeing: the Australian Unity Wellbeing Index. Social Indicators Research.

[CR13] De Jong Gierveld J, Van Tilburg T (2006). A 6-item scale for overall, emotional, and social loneliness - confirmatory tests on survey data. Research on Aging.

[CR14] Galeoto G, Sansoni J, Scuccimarri M, Bruni V, De Santis R, Colucci M (2018). A psychometric properties evaluation of the Italian version of the geriatric depression scale. Depression Research and Treatment.

[CR15] Gallardo-Peralta LP, Molina MA, Schettini R (2019). Validation of the Personal Wellbeing Index (PWI) for older Chilean adults. International Psychogeriatrics.

[CR16] Gallardo-Peralta LP, Sánchez-Moreno E, Barrón A, Arias A (2015). Ethnicity, social support, and depression among elderly Chilean people. The Journal of Psychology: Interdisciplinary and Applied.

[CR17] Gallardo-Peralta LP, Sánchez-Moreno E, Rodríguez-Rodríguez V (2019). Strangers in their own world: exploring the relation between cultural practices and the health of older adults in native communities in Chile. British Journal of Social Work.

[CR18] Gavilan V, Vigueras PC, Madariaga E, Parra MG (2018). Intercultural aspects of health. A critical analysis of health policies aimed at the Aymara people of northern Chile. Interciencia.

[CR19] Gracia E, Herrero J, Musitu G (2002). Evaluación de recursos y estresores psicosociales en la comunidad.

[CR20] Grundy EM, Albala C, Allen E, Dangour AD, Elbourne D, Uauy R (2012). Grandparenting and psychosocial health among older Chileans: a longitudinal analysis. Aging & Mental Health.

[CR21] Guerrero B (1995). Medicina andina y medicina pentecostal en los aymaras del norte grande de Chile: del yatiri al pastor. Chungara.

[CR22] Herrero J, Gracia E (2005). Redes sociales de apoyo y ajuste biopsicosocial en la vejez: Un análisis comparativo en los contextos comunitario y residencial. Intervención Psicosocial.

[CR23] Hoyl T, Valenzuela E, Marín PP (2000). Depression in the aged: preliminary evaluation of the effectiveness, as an screening instrument, of the 5-item version of the Geriatric Depression Scale. Revista Médica de Chile.

[CR24] Hunter E (2014). Mental health in Indigenous settings. Challenges for clinicians. Australian Family Physician.

[CR25] Juniper EF, Guyatt GH, Jaeschke R, Spilker B (1996). How to develop and validate a new health-related quality of life instrument. Quality of life and pharmacoeconomics in clinical trials.

[CR26] Kulathunga, M., Umayal,S., Somaratne,S., Srikanth, S., Kathriarachchi, S., & De Silva, K. R. D. (2010). Validation of the Geriatric Depression Scale for an elderly Sri Lankan clinic population. Indian Journal of Psychiatry, 52(3): 254–256. doi: 10.4103/0019-5545.70979 10.4103/0019-5545.70979 PMC2990826 21180411

[CR27] Linacre JM (1994). Sample size and item calibration or person measure stability. Rasch Measurement Transactions.

[CR28] Luo, H., Lou, V. W. Q., Chen, C., & Chi, I. (2018). The effectiveness of the positive mood and active life program on reducing depressive symptoms in long-term care facilities. *The Gerontologist*. 10.1093/geront/gny120. 10.1093/geront/gny120 30295729

[CR29] Martínez de la Iglesia J, Onís Vilches MC, Dueñas Herrero R, Albert Colomer C, Aguado Taberné C, Luque Luque R (2002). Versión española del cuestionario de Yesavage abreviado (GDS) para el despistaje de depresión en mayores de 65 años: adaptación y validación. Medifam.

[CR30] Martinez-Martin, P., & Forjaz, M. J. (2012). How to evaluate validation data. En C. Sampaio, C. G. Goetz, & A. Schrag (Eds.), Rating scales in Parkinson’s disease (pp. 16-41). New York: Oxford University Press.

[CR31] Massai, P., Colalelli, F., Sansoni, J., Valente, D., Tofani, M., & Fabbrini, G. … Galeoto, G. (2018). Reliability and validity of the Geriatric Depression Scale in Italian subjects with Parkinson’s disease. *Parkinsons Disease*, *7347859*. 10.1155/2018/7347859. 10.1155/2018/7347859 PMC6093010 30155239

[CR32] McHorney CA, Tarlov AR (1995). Individual-patient monitoring in clinical practice: are available health status surveys adequate?. Quality of Life Research.

[CR33] McIntyre C, Harris MG, Baxter AJ, Leske S, Diminic S, Gone JP (2017). Assessing service use for mental health by Indigenous populations in Australia, Canada, New Zealand and the United States of America: a rapid review of population surveys. Health Research Policy and Systems.

[CR34] Mella R, Alvear M, Carrillo B, Caire V (2003). Evaluation of mental and communication functions in mapuche and non mapuche elderly subjects in rural communities in Southern Chile. Revista Médica de Chile.

[CR35] Ministry of Social Development. (2017). Encuesta Nacional de Caracterización Socioeconómica, CASEN. Resource document. Observatorio Social. http://observatorio.ministeriodesarrollosocial.gob.cl/casen-multidimensional/casen/casen_2017.php. Accessed 7 Feb 2019.

[CR36] Omachi TA, Katz PP, Yelin EH, Gregorich SE, Iribarren C, Blanc PD, Eisner MD (2009). Depression and health-related quality of life in chronic obstructive pulmonary disease. The American Journal of Medicine.

[CR37] Oyarce A, Pedreros M (2007). Perfil Epidemiológico Básico de la Población Aymara del Servicio de Salud Iquique.

[CR38] Pacheco JA (2012). Structure and social change in indigenous societies in Latin America. The relation between Mapuche society and Chilean state. Desacatos.

[CR39] Pallant JF, Tennant A (2007). An introduction to the Rasch measurement model: an example using the Hospital Anxiety and Depression Scale (HADS). The British Journal of Clinical Psychology.

[CR40] Petribu, K., Lima, F., Brotto, S., Leao, D., Arruda, M., & Araujo,S (2017). Depressive symptoms in elderly indigenous people of the Xukuru do Ororuba ethinic group, Brazil. European Neuropsychopharmacology, 27(4), S883-S884. doi: 10.1016/S0924-977X(17)31579-1.

[CR41] Pocinho MTS, Farate C, Dias CA, Lee TT, Yesavage JA (2009). Clinical and psychometric validation of the Geriatric Depression Scale (GDS) for Portuguese elders. Clinical Gerontologist.

[CR42] Power M, Quinn K, Schmidt S, WHOQOL-OLD Group (2005). Development of the WHOQOL-old module. Quality of Life Research.

[CR43] Roh S, Burnette CE, Lee KH, Lee Y-S, Easton SD (2016). Risk and protective factors for depressive symptoms among indigenous older adults: intimate partner violence (IPV) and social support. Journal of Gerontological Social Work.

[CR44] Ruipérez I, Fernández-Ballesteros R (2009). Escalas de valoración en contextos geriátricos. *Gerontología social*.

[CR45] Sandoval FA, Tamiya N, Lloyd-Sherlock P, Noguchi H (2016). Relation of depression with health behaviors and social conditions of dependent community-dwelling older persons in the Republic of Chile. International Psychogeriatrics.

[CR46] Sinclair VG, Wallston KA (2004). The development and psychometric evaluation of the Brief Resilient Coping Scale. Assessment.

[CR47] Tennant A, Conaghan PG (2007). The Rasch measurement model in rheumatology: what is it and why use it? When should it be applied, and what should one look for in a Rasch paper?. Arthritis and Rheumatism.

[CR48] Turner JA, Ersek M, Kemp C (2005). Self-efficacy for managing pain is associated with disability, depression, and pain coping among retirement community residents with chronic pain. The Journal of Pain.

[CR49] Urzúa A, Navarrete M (2013). Factor analysis of abbreviated versions of the WHOQoL-Old in Chilean older people. Revista Médica de Chile.

[CR50] Ventura-León JL, Caycho T (2017). Validez y fiabilidad de la Escala de Soledad de Jong Gierveld en jóvenes y adultos peruanos. PSIENCIA. Revista Latinoamericana de Ciencia Psicológica.

[CR51] Walker RJ, Campbell JA, Dawson AZ, Egede LE (2019). Prevalence of psychological distress, depression and suicidal ideation in an indigenous population in Panamá. Social Psychiatry and Psychiatric Epidemiology.

